# The longitudinal relationships between pain severity and disability versus health-related quality of life and costs among chronic low back pain patients

**DOI:** 10.1007/s11136-019-02302-w

**Published:** 2019-09-17

**Authors:** E. N. Mutubuki, Y. Beljon, E. T. Maas, F. J. P. M. Huygen, R. W. J. G. Ostelo, M. W. van Tulder, J. M. van Dongen

**Affiliations:** 1 Department of Health Sciences, Faculty of Science, Vrije University Amsterdam, Amsterdam Movement Sciences, Amsterdam, The Netherlands; 2 School of Population and Public Health, University of British Columbia, Vancouver, Canada; 3 Department of Anesthesiology, Centre of Pain Medicine, ErasmusMC, Rotterdam, The Netherlands; 4 Department of Epidemiology and Biostatistics, VU University Medical Center, Amsterdam Movement Sciences, Amsterdam, The Netherlands; 5 Department of Physiotherapy & Occupational Therapy, Aarhus University Hospital, Aarhus, Denmark

**Keywords:** Pain, Disability, Health-related quality of life, Societal costs, Longitudinal analysis, Low back pain

## Abstract

**Purpose:**

Previous studies found higher levels of pain severity and disability to be associated with higher costs and lower health-related quality of life. However, these findings were based on cross-sectional data and little is known about the longitudinal relationships between pain severity and disability versus health-related quality of life and costs among chronic low back pain patients. This study aims to cover this knowledge gap by exploring these longitudinal relationships in a consecutive cohort.

**Methods:**

Data of 6316 chronic low back pain patients were used. Measurements took place at 3, 6, 9, and 12 months. Pain severity (Numeric pain rating scale; range: 0–100), disability (Oswestry disability index; range: 0–100), health-related quality of life (EQ-5D-3L: range: 0–1), societal and healthcare costs (cost questionnaire) were measured. Using linear generalized estimating equation analyses, longitudinal relationships were explored between: (1) pain severity and health-related quality of life, (2) disability and health-related quality of life, (3) pain severity and societal costs, (4) disability and societal costs, (5) pain severity and healthcare costs, and (6) disability and healthcare costs.

**Results:**

Higher pain and disability levels were statistically significantly related with poorer health-related quality of life (pain intensity: − 0.0041; 95% CI − 0.0043 to − 0.0039; disability: − 0.0096; 95% CI − 0.0099 to − 0.0093), higher societal costs (pain intensity: 7; 95% CI 5 to 8; disability: 23; 95% CI 20 to 27) and higher healthcare costs (pain intensity: 3; 95% CI 2 to 4; disability: 9; 95% CI 7 to 11).

**Conclusion:**

Pain and disability were longitudinally related to health-related quality of life, societal costs, and healthcare costs. Disability had a stronger association with all outcomes compared to pain.

## Introduction

Low back pain (LBP) is a highly prevalent health complaint. In 2015, the global point prevalence of activity-limiting LBP was estimated at 7.3%, implying that about 540 million people worldwide were affected by LBP at that moment in time [1]. Previous studies reported the lifetime-prevalence of LBP to range from 60 to 85% [2–5]. This indicates that people have a high probability of developing an LBP episode at any time during their life. In the upcoming decades, the aging of the population will likely lead to an increased prevalence of LBP as well as an increased number of patients whose pain persists for a period longer than 3 months (also defined as chronic LBP) [6, 7].

Chronic LBP is associated with high pain levels, significant physical limitations, poorer prognosis, lower health-related quality of life and disability [3, 8–10]. Around 57 million years lived with disability were found to be associated with LBP in 2016, and these have increased by more than 50% since 1990 [11]. Chronic LBP patients report quality of life scores that are comparable to those individuals with a life-threatening diagnosis [12]. Even though only 10–15% of LBP patients develop chronic LBP, research suggests that chronic LBP is responsible for the majority of LBP-related societal costs [6]. In the Netherlands, these LBP-related societal costs were estimated to be as high as 3.5 billion euros in 2007, which equals about 0.6% of the Dutch gross national product (GNP) [6]. In the United States, the estimated annual total societal cost of LBP was estimated at 100 billion dollars [13, 14]. Absenteeism, early retirement, and a loss of productivity while being at work are the most important drivers of these societal costs [15].

Previous studies found a higher level of pain severity and/or disability to be related to higher costs and a lower health-related quality of life [10, 16–19]. A study by Horng et al. for example, reported significant correlations between pain intensity and disability and health-related quality of life [17, 18]. Long lasting, persisting pain and functional limitations that LBP patients experience can cause disability and interfere with their quality of life [17, 20]. Chiarotto et al. reported a positive correlation between pain severity, as measured using a Numeric Rating Scale (NRS), and disability and a negative correlation between pain severity, as measured using the brief pain inventory-pain severity, and health-related quality of life [21]. Sadosky et al. found that an increasing pain severity level was associated with higher indirect costs (i.e., productivity-related costs), direct costs (i.e., healthcare costs), and societal costs amongst Japanese LBP patients [19].

Previous studies on the relation between pain severity and disability versus health-related quality of life and healthcare and societal costs among chronic LBP patients were cross-sectional in nature [19]. This means that they explored whether pain severity and/or disability were associated at a certain point in time with health-related quality of life and/or healthcare and societal costs. Such cross-sectional studies do not provide insight into whether individual *changes* in one variable (e.g., pain severity) are related to individual *changes* in another (e.g., costs). Such relationships can only be studied using a longitudinal study design, in which both variables are measured and compared over time [22].

This study aims to cover this knowledge gap by exploring the longitudinal relationships between pain severity and disability versus health-related quality of life, healthcare and societal costs among chronic LBP patients. Based on previous cross-sectional research, we expect that higher pain and disability are associated with reduced health-related quality of life (negative longitudinal relationship) and higher healthcare and societal costs (positive longitudinal relationship). Next to providing valuable information for clinical practice, information on the longitudinal relationships between pain severity and disability versus health-related quality of life and costs amongst chronic LBP patients, could provide valuable input for health economic modeling studies in the area of chronic LBP.

## Methods

### Study population and design

Data collected during the MinT (minimal invasive treatment) study [23] were used to explore the longitudinal relationships between pain severity and disability versus health-related quality of life and costs among chronic LBP patients. The MinT study was conducted in the Netherlands, and consisted of three randomized controlled trials and an observational study. The overall aim of the MinT study was to assess the effectiveness and cost-effectiveness of adding minimal interventional procedures to a standardized treatment program, compared with a standardized treatment program alone [23, 24]. A detailed description of the MinT study can be found elsewhere [23]. In the present study, only data of *chronic* LBP patients participating in the observational branch of the MinT study were used (i.e., patients experiencing LBP symptoms for more than 12 weeks). In order to be eligible to participate in the observational study, and thus to be included in the present study, patients had to be aged between 18 and 70 years, referred to a pain clinic with suspected chronic mechanical LBP and without improvement of symptoms after conservative treatment [23]. The observational study monitored patients who did not want to, or were not eligible, to participate in the aforementioned randomized controlled trials [23].

### Outcome measures

#### Dependent variables: health-related quality of life, societal costs, and healthcare costs

Three dependent variables were used in this study, all of which were measured at 3, 6, 9, and 12-month follow-up. Health-related quality of life was also measured at baseline, whereas healthcare and societal costs were not. To improve comparability across the analyses, only follow-up measurement values were used for assessing the longitudinal relationships.

1. *Health-related quality of life* Health-related quality of life was measured using the EQ-5D-3L. The EQ-5D-3L is a health-related quality of life scale that has previously been found to be responsive amongst chronic LBP patients [25]. The EQ-5D-3L consists of five dimensions of health, including mobility, self-care, daily activities, pain/discomfort, and anxiety/depression, each with three levels of severity. The participants’ EQ-5D-3L scores were converted into utility values using the Dutch tariff [26]. Utility values are preference weights, indicating a person’s value or desirability of a certain health state on a scale anchored at 0 (equal to death) and 1 (equal to full health) [27].
2. *Societal costs* Comprised in societal costs were healthcare, informal care, unpaid productivity and work absenteeism costs. Resource use was measured using cost questionnaires [28]. Healthcare use included the use of primary care (e.g., visits to a general practitioner or physiotherapist) and secondary care (e.g., visits to a medical specialist or pain clinic). Data from the updated Dutch Manual of Costing were used to value costs of common healthcare interventions, such as appointments with a general physician and a physical therapist [29]. Costs of less common interventions were estimated using an average of five quotes from various practitioners across the country and/or pricelists of professional organizations. Informal care and unpaid productivity were valued using a recommended Dutch shadow price [29]. To measure work absenteeism, the Productivity and disease Questionnaire (PRODISQ) was used [30]. Absenteeism costs were estimated in accordance with the friction cost approach and using gender-specific price weights provided by the updated Dutch Manual of Costing [29]. All cost categories were measured with 3-month recall periods [28].
3. *Healthcare costs* Comprised in healthcare costs were primary and secondary healthcare costs. The measurement and valuation of healthcare costs has been outline above.

#### Independent variables: pain severity and disability

Two independent variables were used in this study, both of which were measured at baseline, 3, 6, 9, and 12-month follow-up:

1. *Pain intensity* Pain severity was measured using the NPRS (range 0—no pain to 10—worst pain imaginable). Scores were transformed to a 0–100 scale to improve the interpretation and comparability of outcomes. Several studies concluded that the validity and sensitivity of the NPRS was appropriate for measuring pain in chronic LBP patients [31, 32]. A clinically meaningful change for people with LBP on the NPRS was previously found to be two (equalling 20 on the 0–100 scale) [33].
2. *Disability* Disability was measured using the Oswestry Disability Index (ODI: range 0—no disability to 100—maximum disability possible). The ODI is a commonly used outcome measure amongst LBP patients [34–37] and is reported to be a valid, reliable and responsive hence suitable as a clinical measure [34]. A clinically meaningful change for people with LBP on the ODI was previously found to be ten points on the 0–100 point ODI [38].

The ODI and NPRS are both part of the core outcome set recommended for LBP [39].

### Potential confounding factors

Potential confounding factors included were based on literature [40] and measured at baseline. These included:

- Patient expectations (Credibility/Expectancy Questionnaire [CEQ] [41]; range 0—least credibility/expectancy to 100—more credibility/expectancy).
- Pain severity (Numeric Pain Rating Scale [NPRS]; range 0—no pain to 100—worst pain imaginable) [33]. For the purpose of this study, scores were transformed to 0–100. (In the analyses in which disability and health-related quality of life were included).
- Disability (Oswestry Disability Index [ODI]; range 0—no disability to 100—maximum disability) [36, 42]. (In the analyses in which pain and health-related quality of life were included).
- Health-related quality of life (EuroQol [EQ-5D-3L]; range 0—equal to death 1—equal to full health) [43].
- General health—mental component score and physical component score (Rand-36 [Rand-36]; scores range 0—lowest general health to 100—highest general health) [44–46]. The two component scores were assessed for being a confounding variable separately.
- Impact of pain experience (Multidimensional Pain Inventory [MPI]; range 0—least/best to 100—most/worst) [47, 48].
- Education level (low/moderate/high). Low-indicates, no education, primary level education, lower vocational and lower secondary education, moderate-indicates higher secondary education or undergraduate, high-indicates tertiary education university or postgraduate).
- Body Mass Index ([BMI], weight in kg/(height in meters)^2^).
- Employment (yes/no).
- Recurrent complaints (yes/no).
- Age (years).
- Gender (male/female).
- Nationality (Dutch/non-Dutch).
- Smoking (yes/no).
- Type of health care insurance (basic/additional).
- Region of residence (south/north/east/west).
- Married/living together yes/no).
- Diagnosis (sacroiliac joint (SI)/facet/disc/combined/unclear).

### Statistical analysis

The patients’ baseline characteristics were descriptively summarized. Missing data were handled using multiple imputation to avoid possible bias due to selective drop-out of participants [49]. Imputations were performed using the Multiple Imputation by Chained Equations algorithm with predictive mean matching [50]. The imputation model included all available potential confounders, pain intensity, disability, health-related quality of life, and cost values.

For answering the research question, linear generalized estimating equation (GEE) analyses were performed. A GEE analysis is a so-called sophisticated longitudinal data analysis technique, in which the relationship between the variables in the model (e.g., pain severity and societal costs) at different time points (i.e., 3, 6, 9, and 12 months) is analyzed simultaneously. Herewith, the estimated regression co-efficient reflects the longitudinal relationship between the dependent variable (e.g., societal costs) and the independent variable(s) (e.g., pain severity), using all available data, and thus providing an indication of whether *changes* in the dependent variable are related to *changes* in the independent variable [22] within and between participants over different measurement time points. Six separate longitudinal relationships were assessed between: (1) pain severity and health-related quality of life, (2) disability and health-related quality of life, (3) pain severity and societal costs, (4) disability and societal costs, (5) pain severity and healthcare costs, and (6) disability and healthcare costs. Longitudinal relationships (1) and (2) were explored with a Gaussian distribution and an identity link. Longitudinal relationships (3) to (6) were explored with a gamma distribution and an identity link. The gamma distribution was chosen to take into account the right skewed nature of cost data. In all of the analyses, an exchangeable correlation structure was assumed. First, crude analyses were performed that solely included the dependent and the independent variables. Second, adjusted analyses were performed that also included potential confounding factors. Variables that changed the regression co-efficient by more than 10% were deemed confounders and were included in the model. All analyses were performed in Stata (version 14 SE, Stata Corp). Statistical significance was set on *p* < 0.05.

## Results

### Participants

**Fig. 1:**
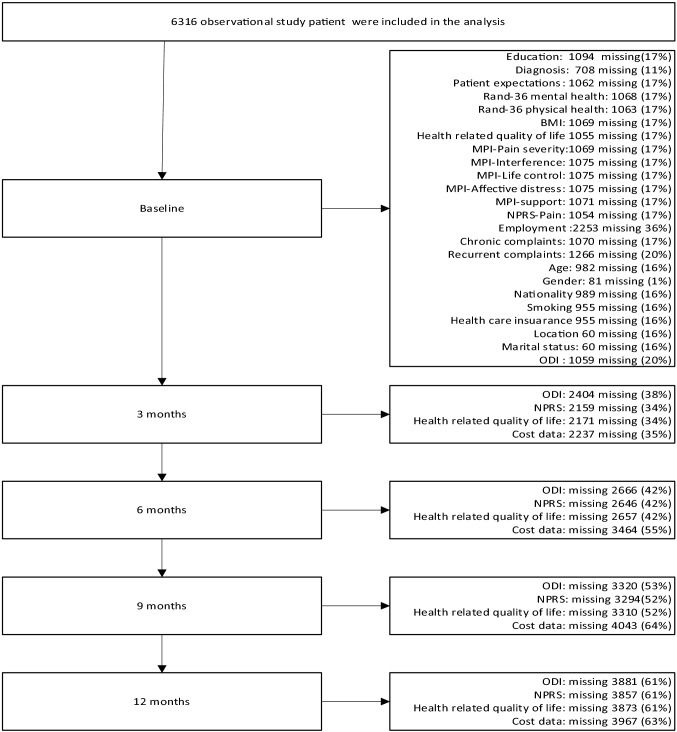
Flowchart of missing data at each follow-up moment

Data from 6316 chronic LBP patients were analyzed in the present study. Of them, the majority were female (66%), overweight (67%), Dutch (95%), had a low level of education (56%), had a mean age of 57 years and more than half were unemployed (59%) (Table 1). Cost data had the highest percentage of missing data and most data were missing at 9-month follow-up. A detailed description of the percentages of missing data per outcome and per time point can be found in Fig. 1.

**Table 1:** Patient characteristics

Participant characteristic	All patients (n = 6316)
Age (years) [mean (SD)]	57.2 (13,4)
Gender [*n* (%)]	
Female	4142 (66)
Male	2093 (34)
BMI [*n* (%)]	
BMI < 18.5 (underweight)	37 (1)
BMI ≥ 18.5 < 25 (normal weight)	1687 (32)
BMI ≤ 25 < 30 (overweight)	2060 (39)
BMI ≥ 30 (obese)	1463 (28)
Smoking [*n* (%)]	
Yes	1413 (26)
No	3920 (73)
Educational level [*n* (%)]	
Low (no education, primary level education, lower vocational and lower secondary education)	2925 (56)
Moderate (higher secondary education or undergraduate)	1467 (28)
High (tertiary, university level, postgraduate)	830 (16)
Living together with a partner [*n* (%)]	
Yes	4663 (75)
No	1593 (26)
Nationality [*n* (%)]	
Dutch	5049 (95)
Non-Dutch:	278 (5.2)
Surinamese	*21 (0.4)*
Antillean/Aruban	*22 (0.4)*
Turkish	*63 (1)*
Moroccan	*42 (1)*
Other	*130 (2.4)*
Region in the Netherlands [*n* (%)]	
South	2029 (32)
North	1165 (19)
East	1280 (20)
West	1782 (28)
Employment [*n* (%)]	
Yes	1687(42)
No	2376 (59)
Recurrent low back pain [*n* (%)]	
Yes	3174 (63)
No	1876 (37)
Diagnosis-source of pain [*n* (%)]	
1 = SI	1864 (33)
2 = Facet	2269 (41)
3 = Disc	18 (0.3)
4 = Combined	1391 (25)
5 = Unclear	66 (1)
Patients expectations	
Credibility [mean (SD)] range 0–100	77.1 (17.5)
Expectancy [mean (SD)] range 0–100	57.8 (17.3)
Rand-36	
Mental [mean (SD)] range 0–100	22.6 (5)
Physical [mean (SD)] range 0–100	18.5 (4)
Health-related quality of life(utility) [mean (SD)] range 0–100	48 (29)
MPI [mean (SD)] range per subscale 0–100	
Pain severity	22.6 (5.7)
Interference with daily activities	5.8 (1.9)
Life control	21.2 (6.3)
Affective distress	15.4 (4.6)
Support	28.6 (7.6)
Type of health care insurance [*n* (%)]	
Basic insurance	633 (12)
Comprehensive (basic + additional cover)	4630 (86)
I don’t know	55 (1)
ODI functional disability [mean (SD)] range 0–100	11.1 (9)
Pain severity [mean (SD)] range 0–100	73 (16)

Percentages have been rounded off hence values a bit less than 100% and a bit more that 100%

Scores for MPI, Rand 36, patient expectations, health-related quality of life were transformed to a range of 0–100 to enable comparability. Diagnosis was based on patient history and physical examination

*ODI* Oswestry disability index, *MPI* multidimensional pain inventory

### Disability

Table 2 shows the results from the longitudinal analyses between disability and health-related quality of life, societal costs and healthcare costs. Disability and health-related quality of life had a statistically significant negative longitudinal relationship (B: − 0.0096; 95% CI − 0.0099 to − 0.0093). As none of the possible confounding factors changed the regression co-efficient by more than 10% an adjusted model was not required. The crude analysis using disability and societal costs suggested a significant positive longitudinal relationship (B: 25; 95% CI 20 to 29). After adjusting for confounding, the identified longitudinal relationship between disability and societal costs remained statistically significant (B: 23; 95% CI 20 to 27). A significant positive longitudinal relationship was also observed in both the crude (B: 10; 95% CI 9 to 12) and adjusted (B: 9; 95% CI 7 to 11) analyses using disability and healthcare costs.

**Table 2:** Longitudinal analyses between disability, societal costs, healthcare costs and health-related quality of life

Results disability	Crude			Adjusted		
Disability ODI (0–100)	Beta	95% CI		Beta	95% CI	
		Lower bound	Upper bound		Lower bound	Upper bound
Total costs-societal perspective^a^	24	20	29	17	14	20
Total costs-health care perspective^b^	10	9	12	8	6	9
Health-related quality of life^c^	− 0.0096	− 0.0099	− 0.0093			

Scores for health-related quality of life were transformed to a range of 0–100 to enable comparability

^a^Adjusted for health-related quality of life, physical health, MPI life control, MPI_Inteference, MPI_Pain severity, mental health, disability

^b^Adjusted for physical health (SF-36), pain impact experience (MPI interference), health-related quality of life (EQ-5D)

^c^No confounding factors; none of the confounders changed the regression co-efficient by more than 10%

### Pain

Table 3 shows the results from the longitudinal analyses between pain and health-related quality of life, societal costs and healthcare costs. A significant negative longitudinal relationship was observed between pain and health-related quality of life. After adjusting for confounding, the results still suggested a significant negative longitudinal relationship between pain and healthcare costs (B: − 0.0041; 95% CI − 0.0043 to − 0.0039). The crude analyses using pain and societal costs suggested a significant positive longitudinal relationship (B: 8; 95% CI 6 to 10). After adjusting for confounding, the adjusted analysis also suggested a significant positive longitudinal relationship between pain and societal costs (B: 7; 95% CI 5 to 8). Pain and healthcare costs also had a significant positive longitudinal relationship in both the crude (B: 3; 95% CI 2 to 5) and adjusted analysis (Beta = B: 2; 95% CI 2 to 4).

**Table 3:** Longitudinal analyses between pain, societal costs, healthcare costs and health-related quality of life

Results pain	Crude			Adjusted		
Pain (0–100)	Beta	95% CI		Beta	95% CI	
		Lower bound	Upper bound		Lower bound	Upper bound
Total costs-societal perspective^a^	8	6	10	5	4	6
Total costs-health care perspective^b^	3	2	5	2	2	3
Health-related quality of life^c^	− 0.0041	− 0.0043	− 0.0038			

^a^Adjusted for health-related quality of life, physical health, MPI life control, mental health, disability

^b^Adjusted for MPI life control, MPI_Inteference, MPI_Pain severity, health-related quality of life, physical health (SF-36), mental health

^c^No confounding factors; none of the confounders changed the regression co-efficient by more than 10%

## Discussion

### Main findings

This study found pain severity and disability both to have a statistically significant negative longitudinal relationship with health-related quality of life, and a statistically significant positive longitudinal relationship with societal as well as healthcare costs. In GEE, regression co-efficients have a double interpretation resulting in a pooled co-efficient of a within-subject and a between-subject effect [22]. Interpreting these regression co-efficients in terms of practical relevance indicates that a 1-point *increase* in disability, for example, is related to a 0.0096 point *decrease* in health-related quality of life (range 0–1), 23 euros *increase* in societal costs and 9 euros *increase* in healthcare costs per 3 months. A clinically relevant *increase* in disability (defined as a 10 point increase on the 0–100 point ODI) [38] is thus associated with a *decrease* in health-related quality of life by 0.096 points (range 0–1), and an *increase* in societal as well healthcare costs by 230 and 90 euros per 3-month period. Thus, the potential costs savings associated with relevant improvements in pain and disability are tremendous in a highly prevalent disorder such as low back pain. Moreover, a clinically relevant increase in disability was found to be longitudinally related to a more than clinically relevant increase in health-related quality of life, which was previously found to be equal to an increase of 0.057 or more [51, 52]. For pain intensity, the associated decrease in health-related quality of life was slightly smaller than the established minimal clinically relevant difference for health-related quality of life (i.e., 0.0041 vs. 0.059).

All of our findings were in line with our expectation that pain and disability would have a statistically significant negative relationship with health-related quality of life and a statistically significant positive longitudinal relationship with societal costs and healthcare costs. Also, it is noteworthy that the impact of pain on health-related quality of life and costs was found to be about 2.5 times smaller than the impact of disability on health-related quality of life and costs even though patients had high baseline scores of pain and relatively ‘lower scores’ on disability. This might suggest that it is not the level of pain severity that has a strong association with an individual’s health-related quality of life and/or costs but the way in which an individual’s pain influences his or her daily activities. However, further research is needed to confirm this.

### Comparison with literature

To the best of our knowledge, no studies have explored the longitudinal relationships between pain severity and disability versus health-related quality of life and costs. Nonetheless, a cross-sectional study by Sadosky et al. found an increasing pain severity to be related with a worsening health-related quality of life as well as increased healthcare and societal costs. This is in line with the findings of the present study. In contract to the present study, however, Sadosky et al. also included presenteeism costs (i.e., costs related to reduced productivity while being at work) and their study was conducted among acute as well as chronic LBP patients instead of chronic LBP patients only. Like the present study, a study of Stefane et al. found a significant negative association between pain and disability versus health-related quality of life. In line with the current study, Stefane et al. found health-related quality of life to be more strongly associated to disability than to pain. Unlike the present longitudinal study, the study of Stefane et al. was cross-sectional in nature and disability was measured using the Ronald–Morris 24 items questionnaire [53] instead of using the ODI.

In our study, disability was found to have about 2.5 times higher impact on health-related quality of life, societal costs, and healthcare costs. Our reasoning that, this might suggest that, it is not the level of pain severity that has a strong association with an individual’s health-related quality of life and/or costs but the way in which an individual’s pain influences his or her daily activities is supported by a study of Horng et al. In their study, Horng et al. reported that pain persistence and limitation of activities for daily living had more influence on a patient’s health-related quality of life compared to pain severity alone in both acute and chronic LBP patients [17]. Our reasoning is further supported by Lame et al. who reported pain catastrophizing as the most important predictor of individual health-related quality of life in a heterogeneous group of chronic pain patients. In their study pain catastrophizing had the strongest association with individuals health-related quality of life compared to pain severity and chronic LBP patients had the lowest quality of life [54]. Pain catastrophizing is generally defined as excessive negative orientation towards pain/noxious stimuli [55, 56]. High levels of pain catastrophizing were associated with disability, poor outcomes, and pain severity for patients with LBP [56–58].

### Strengths and limitations

Strengths of the present study include that it is the first study to use a longitudinal design to explore whether relationships exist between pain severity and disability versus health-related quality of life and costs. In addition, the large cohort of observed patients with chronic LBP patients (*n *= 6316) greatly increases the power of this study. Another advantage is the use of imputation methods to deal with missing data thereby avoiding complete-case analysis, which would have significantly reduced the study’s power and precision. Multiple imputation is the preferred statistical method for dealing with missing, particularly when costs are involved [49].

Limitations of the present study include the absence of presenteeism costs in the analyses, whereas presenteeism more than absenteeism is reported to be disproportionately affected by pain [16]. As the results of Sadosky et al. who did include presenteeism costs, were in line with those of the present study, we do not expect the absence of presenteeism costs to have greatly biased our conclusion. Nonetheless, future studies should include presenteeism costs to give a more accurate representation of true costs related to lost productivity. Second, there is an over representation of females (66.4%) in the present study, in contrast with the percentage of women with LBP in the Netherlands (56%). This could have resulted in an underestimation of costs since men earn more than women [59] and tend to use more healthcare for LBP [60]. Nonetheless, as stratified post hoc analyses indicated that, except for one unadjusted analyses, all longitudinal relationships were statistically significant amongst men and women with similar beta co-efficients (Appendix 1), we do not expect the overrepresentation of women to have severely biased our results and conclusions. Future studies should include a larger representation of males, reflecting the 44% of males suffering from chronic LBP, to enable better generalizability of our results. Third, although mainly valid and reliable questionnaires were used, the self-reported nature of the questionnaires might have caused recall and or social desirability bias. We tried limiting the recall bias by minimizing the recall period to 3 months [29]. As it seems unlikely that recall bias or the degree to which participants gave socially desirable answers systematically differed over time, it is not expected that self-report biased the results. Fourth, lack of comorbidity factors, which could have been potential confounding factors, could have led to underestimation of costs and the impact on health-related quality of life, since confounding could not controlled for. Fifth, in the present study, the EQ-5D-3L was used to measure health-related quality of life, whereas since the inception of the MinT study [23], an updated five level version of the EQ-5D has been published [61]. However, as both have previously found to be valid means to measure health-related quality of life, we do expect our reliance on the EQ-5D-3L to have biased our results [62]. Also, even though GEE analysis offers an efficient means to analyze the longitudinal relationship between variables, its results may heavily depend on the assumptions made. That is, with GEE analysis, the adjustment for time is carried out by assuming a priori a certain “working” correlation structure for the repeated measurements. Even though GEE analysis is assumed to be robust against a wrong choice of correlation structure, evidence suggests that results may differ extensively across correlation structures [22]. Based on the recommendations of Twisk et al. we assumed an “exchangeable” correlation structure, in which correlations between subsequent measurements are assumed to be equal irrespective of the length of the time intervals [22]. To assess the robustness of the current findings to the choice of correlation structure, we performed a post hoc analysis with an “unstructured” correlation structure, in which no particular structure is assumed and all possible correlations between repeated measurements have to be estimated [22]. As the results of the post hoc analysis are in line with those of the main analysis (“Appendix 1”), we consider the current findings to be robust against the choice of correlation structure.

### Implications for practice and research

Our findings indicate that the potential costs savings associated with relevant improvements in pain and disability are tremendous in a prevalent disorder such as LBP. A clinical improvement in disability, 10 points on the 0–100 point ODI [38], will result in potential savings of 230 per LBP patient per 3 months. Our study also provides some preliminary evidence, that is, disability is more associated with higher societal and healthcare costs and poorer health-related quality of life, than pain severity. Further research into this topic is warranted, but for now these findings at least suggest that focussing initiatives and interventions on disability more that pain severity may improve patient outcomes, i.e., health-related quality of life and costs. Also, the aim of the present study was to explore the separate relationships of pain and disability with healthcare costs, societal costs, and health-related quality of life. Therefore, the combined influence of pain severity and disability on costs and health-related quality of life was not explored, neither were the potential interactions between pain and disability. This should be explored further in future research.

## Conclusion

The present study showed that both pain severity and disability are longitudinally related to health-related quality of life, societal costs, and healthcare costs. Disability had a stronger association with all outcomes compared to pain, suggesting that it is not the level of pain severity that influences the height of an individual’s health-related quality of life and costs, but the way in which an individual’s pain influences his or her daily activities.

## Appendix 1

See Tables 4, 5, 6, and 7.

**Table 4:** Longitudinal analyses between disability, societal costs, healthcare costs, and health-related quality of life among men

Results disability	Crude			Adjusted		
Disability ODI (0–100)	Beta	95% CI		Beta	95% CI	
		Lower bound	Upper bound		Lower bound	Upper bound
Total costs-societal perspective^a^	23	20	25	15	12	18
Total costs-health care perspective^b^	9	7	11	5	4	7
Health-related quality of life^c^	− 0.0094	− 0.0098	− 0.0091			

Scores for health-related quality of life were transformed to a range of 0–100 to enable comparability

^a^Adjusted for health-related quality of life, physical health, MPI life control, MPI_Inteference, MPI_Pain severity, mental health, disability

^b^Adjusted for physical health (SF-36), pain impact experience (MPI interference), health-related quality of life (EQ-5D)

^c^No confounding factors; none of the confounders changed the regression co-efficient by more than 10%

**Table 5:** Longitudinal analyses between pain, societal costs, healthcare costs, and health-related quality of life among men

Results pain	Crude			Adjusted		
Pain (0–100)	Beta	95% CI		Beta	95% CI	
		Lower bound	Upper bound		Lower bound	Upper bound
Total costs-societal perspective^a^	4	2	7	2	1	4
Total costs-health care perspective^b^	2	− 0.4	3	1	0.2	2
Health-related quality of life^b^	− 0.0039	− 0.0041	− 0.0037			

^a^Adjusted for health-related quality of life, physical health, MPI life control, mental health, disability

^b^Adjusted for MPI life control, MPI_Inteference, MPI_Pain severity, health-related quality of life, physical health (SF-36), mental health

^c^No confounding factors; none of the confounders changed the regression co-efficient by more than 10%

**Table 6:** Longitudinal analyses between disability, societal costs, healthcare costs, and health-related quality of life among women

Results disability	Crude			Adjusted		
Disability ODI (0–100)	Beta	95% CI		Beta	95% CI	
		Lower bound	Upper bound		Lower bound	Upper bound
Total costs-societal perspective^a^	25	21	30	18	15	22
Total costs-health care perspective^b^	11	9	13	9	7	10
Health-related quality of life^c^	− 0.0096	− 0.0099	− 0.0094			

Scores for health-related quality of life were transformed to a range of 0–100 to enable comparability

^a^Adjusted for health-related quality of life, physical health, MPI life control, MPI_Inteference, MPI_Pain severity, mental health, disability

^b^Adjusted for physical health (SF-36), pain impact experience (MPI interference), health-related quality of life (EQ-5D)

^c^No confounding factors; none of the confounders changed the regression co-efficient by more than 10%

**Table 7:** Longitudinal analyses between pain, societal costs, healthcare costs, and health-related quality of life among women

Results pain	Crude			Adjusted		
Pain (0–100)	Beta	95% CI		Beta	95% CI	
		Lower bound	Upper bound		Lower bound	Upper bound
Total costs-societal perspective^a^	8	7	10	6	5	7
Total costs-health care perspective^b^	4	3	4	2	2	3
Health-related quality of life^c^	− 0.0039	− 0.0041	− 0.0038			

^a^Adjusted for health-related quality of life, physical health, MPI life control, mental health, disability

^b^Adjusted for MPI life control, MPI_Inteference, MPI_Pain severity, health-related quality of life, physical health (SF-36), mental health

^c^No confounding factors; none of the confounders changed the regression co-efficient by more than 10%

## Appendix 2

See Table 8 and 9.

**Table 8:** Longitudinal analyses between disability, societal costs, healthcare costs, and health-related quality of life—using a unstructured correlation structure

Results disability	Crude			Adjusted		
Disability ODI (0–100)	Beta	95% CI		Beta	95% CI	
		Lower bound	Upper bound		Lower bound	Upper bound
Total costs-societal perspective^a^	25	21	28	17	14	21
Total costs-health care perspective^b^	11	9	12	8	6	9
Health-related quality of life^c^	− 0.0096	− 0.0099	− 0.0093			

Scores for health-related quality of life were transformed to a range of 0–100 to enable comparability

^a^Adjusted for health-related quality of life, physical health, MPI life control, MPI_Inteference, MPI_Pain severity, mental health, disability

^b^Adjusted for physical health (SF-36), pain impact experience (MPI interference), health-related quality of life (EQ-5D)

^c^No confounding factors; none of the confounders changed the regression co-efficient by more than 10%

**Table 9:** Longitudinal analyses between pain, societal costs, healthcare costs, and health-related quality of life—using an unstructured correlation structure

Results pain	Crude			Adjusted		
Pain (0–100)	Beta	95% CI		Beta	95% CI	
		Lower bound	Upper bound		Lower bound	Upper bound
Total costs-societal perspective^a^	8	7	10	5	4	7
Total costs-health care perspective^b^	4	3	5	2	2	3
Health-related quality of life^a^	− 0.0041	− 0.0043	− 0.0039			

^a^Adjusted for health-related quality of life, physical health, MPI life control, mental health, disability

^b^Adjusted for MPI life control, MPI_Inteference, MPI_Pain severity, health-related quality of life, physical health (SF-36), mental health

^c^No confounding factors; none of the confounders changed the regression co-efficient by more than 10%
